# Enteric coating of tablets containing an amorphous solid dispersion of an enteric polymer and a weakly basic drug: A strategy to enhance *in vitro* release

**DOI:** 10.1016/j.ijpharm.2023.123139

**Published:** 2023-07-25

**Authors:** Hanh Thuy Nguyen, Tu Van Duong, Lynne S. Taylor

**Affiliations:** Department of Industrial and Physical Pharmacy, College of Pharmacy, Purdue University, West Lafayette, IN 47907, United States

**Keywords:** Delamanid, Enteric coating, Amorphous solid dispersion, Weakly basic drug, Enteric polymer, Crystallization, Drug release

## Abstract

Recent work has highlighted that amorphous solid dispersions (ASDs) containing delamanid (DLM) and an enteric polymer, hypromellose phthalate (HPMCP), appear to be susceptible to crystallization during immersion in simulated gastric fluids. The goal of this study was to minimize contact of the ASD particles with the acidic media via application of an enteric coating to tablets containing the ASD intermediate, and improve the subsequent drug release at higher pH conditions. DLM ASDs were prepared with HPMCP and formulated into a tablet that was then coated with a methacrylic acid copolymer. Drug release was studied *in vitro* using a two-stage dissolution test where the pH of the gastric compartment was altered to reflect physiological variations. The medium was subsequently switched to simulated intestinal fluid. The gastric resistance time of the enteric coating was probed over the pH range of 1.6–5.0. The enteric coating was found to be effective at protecting the drug against crystallization in pH conditions where HPMCP was insoluble. Consequently, the variability in drug release following gastric immersion under pH conditions reflecting different prandial states was notably reduced when compared to the reference product. These findings support closer examination of the potential for drug crystallization from ASDs in the gastric environment where acid-insoluble polymers may be less effective as crystallization inhibitors. Further, addition of a protective enteric coating appears to provide a promising remediation strategy to prevent crystallization at low pH environments, and may mitigate variability associated with prandial state that arises due to pH changes.

## Highlights

• Variation of release from delamanid ASDs with an enteric polymer in different physiologically relevant conditions was due to the high crystallization tendency of the drug.
• Enteric coating protected against drug crystallization from ASDs in simulated gastric fluids.
• Enteric coating reduced the impact of pH and food components on *in vitro* drug release from dosage forms containing an ASD of delamanid.

## Graphical abstract

**Figure:**
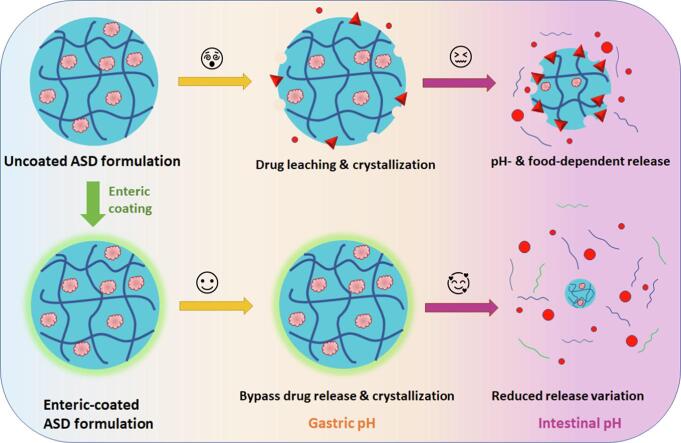

## 1 Introduction

Amorphous solid dispersion (ASD) of a poorly soluble drug and a suitable polymer is a widely used strategy to improve drug solubility, release rate and ultimately absorption, thereby enhancing bioavailability. Preventing drug crystallization during the release process is of critical importance to maintain supersaturation and maximize the driving force for absorption across a membrane. Many polymers, in particular cellulose derivatives, have been found to be effective crystallization inhibitors, delaying nucleation and suppressing crystal growth (Deng et al., 2019, Trasi et al., 2015, Ueda et al., 2014). However, in some cases, polymers found to be effective at inhibiting drug crystallization in the solid formulation during storage, were unable to prevent crystallization upon contact with aqueous media (Schittny et al., 2020, Trasi et al., 2015). In particular, formation of crystals at the surface of the ASD upon initial suspension in aqueous media may be problematic if the crystals are able to undergo additional rapid growth. This was the proposed mechanism for poor release from bicalutamide-copovidone ASDs, where surface crystallization was noted, leading to formation of a crystalline drug boundary layer on the ASD surface (Moseson et al., 2022c).

Weakly acidic polymers, such as poly(acrylic acid), hypromellose acetate succinate (HPMCAS) and hypromellose phthalate (HPMCP), have been frequently used in ASD formulations due to their ability to delay nucleation and inhibit crystal growth (Amponsah-Efah et al., 2020, Deng et al., 2019, Hiew et al., 2022a, Hiew et al., 2022b, Nguyen et al., 2023b, Schram et al., 2016, Van Duong et al., 2022a). HPMCAS was found to effectively prevent various amorphous drugs from assembling into crystalline domains due to limited mobility resulting from the higher glass transition of the ASD imparted by the presence of the polymer, drug-polymer interactions, as well as the polymer dilution effect (Bhugra and Pikal, 2008, Friesen et al., 2008). Anionic polymers have been reported as being effective at inhibiting drug crystallization both in the solid state and in solution for ASD formulations of several drugs (Ting et al., 2015, Trasi et al., 2015, Van Duong et al., 2022a, Xie and Taylor, 2016). However, recent studies suggest that consideration should be given to phase behavior in gastric pH conditions where many anionic polymers such as HPMCAS or HPMCP are insoluble (Monschke and Wagner, 2019, Nguyen et al., 2023b, Wang et al., 2021). A study by Monschke and Wagner (Monschke and Wagner, 2019) of niverapine ASDs showed more extensive drug leaching in the gastric compartment at pH 1 versus pH 4.5. In addition to the impact of pH, the extent of drug release in the gastric compartment may be influenced by drug-polymer ratio, ASD particle size and enteric polymer characteristics (Monschke and Wagner, 2019, Nguyen et al., 2023b, Wang et al., 2021). Importantly, there appears to be a risk for drug crystallization in ASDs with enteric polymers when immersed in simulated gastric fluids, where the polymer is insoluble (Elkhabaz et al., 2019). For posaconazole, ASDs with HPMCAS suspended in fasted state simulated gastric fluid (FaSSGF) pH 1.6 exhibited drug crystallization at high drug loadings (Elkhabaz et al., 2019). Similarly, surface crystallization was found to be maximized at pH 3.0 for ASDs of delamanid (DLM) with either HPMCP or HPMCAS, with a subsequent negative impact on drug release upon transfer to intestinal pH conditions (Nguyen et al., 2023b).

Historically, enteric coating has been applied to modify drug release of oral dosage forms, to protect the active pharmaceutical ingredient (API) from the gastric environment or *vice versa* (Gan et al., 1996, Nguyen et al., 2016, Pavloff et al., 2018, Riekes et al., 2017, Smeets et al., 2020, Tran et al., 2022). Polymers commonly used for enteric coating are cellulose acetate phthalate, HPMCP, HPMCAS, polymethacrylates (marketed as Eudragit®), and polyvinyl acetate phthalate (Siepmann et al., 2006, Thoma and Bechtold, 1999). The pH dissolution threshold of an enteric polymer varies with polymer chemistry, and depends on the number of carboxylic groups as well as other functional groups (Maderuelo et al., 2019). For example, methacrylic acid - ethyl acrylate copolymer (1:1) (also known as Eudragit® L 100–55, Enovik, Germany), can dissolve at a pH above 5.5 (Evonik, 2020), and is available commercially as a formulated powder readily dispersible in water (Acryl EZE® II, Colorcon, US) (Colorcon, 2014). An enteric coating strategy has been applied to ASD formulations in several instances. Riekes and co-workers used Eudragit® L100 for fixed-dose combinations of an ezetimibe and lovastatin ASD with Soluplus (5:5:90, w/w/w) to avoid the formation of the active metabolite of lovastatin in gastric environments (Riekes et al., 2017, Riekes et al., 2016). Enteric-coated formulations were also noted to prevent drug crystallization in acidic media for ASDs of niclosamide with copovidone at a 60% DL (Jara et al., 2022). In another study, enteric-coated darunavir-HPMC ASD nanoparticles were fabricated in a single step using electrospraying (Nguyen et al., 2016). However, to date, enteric coatings have not been investigated for ASDs prepared with an enteric polymer.

The aim of this study was to investigate the impact of an enteric coating on drug release from HPMCP-50 ASDs containing a rapidly crystallizing drug, delamanid, as both the free base form and a salt. Salt formation improved physical stability of ASDs but all formulations showed drug release variations, depending on dissolution medium pH (Nguyen et al., 2023b). It was hypothesized that an enteric coating layer would protect the ASD against unfavorable pH conditions where the polymer is unable to inhibit drug crystallization. This in turn was expected to maintain the dissolution benefits at a higher pH environment where the polymer is both soluble and a better crystallization inhibitor, thereby reducing the drug release variability imparted by immersion in different gastric pH conditions reflective of varying prandial states.

## 2 Experimental section

### 2.1 Materials

Delamanid (DLM) was obtained from Gojira Fine Chemicals, LLC (Bedford Heights, OH) while Deltyba® tablets were manufactured by Otsuka Pharmaceutical Co., Ltd., (Tokyo, Japan). Hydroxypropyl methylcellulose phthalate (HPMCP, P-50 grade) was from Shin-Etsu Chemical Co., Ltd. (Tokyo, Japan). 1,2-Ethanedisulfonic acid dihydrate was supplied by Tokyo Chemical Industry Co. Ltd. (Tokyo, Japan). Croscarmellose sodium (Ac-Di-Sol®) and microcrystalline cellulose pH 101 were sourced from FMC Biopolymer (Newark, DE). Sodium starch glycolate was purchased from JRS Pharma (Posenberg, Germany). Silica, colloidal anhydrous (Aerosil® 200) and Eudragit® L 100-55 were provided by Evonik (Darmstadt, Germany). Magnesium stearate was procured from Spectrum (New Brunswick, NJ). Acryl-EZE® II was obtained from Colorcon (Harleysville, PA). Hydrochloric acid, dichloromethane (DCM), methanol (MeOH), acetone and phosphate salts, maleic acid, sodium hydroxide were supplied by Fisher-Scientific (Pittsburg, PA). Biorelevant simulated gastric and intestinal fluids, including FaSSIF/FaSSGF, FeSSIF-V2 and FEDGAS were purchased from Biorelevant (London, UK).

### 2.2 Methods

#### 2.2.1 Preparation of amorphous solid dispersion

ASDs of DLM free base or DLM salt were prepared at a 25 wt.% drug loading with HPMCP (P-50 grade). DLM salt ASDs were prepared *in situ* by adding acidic counterion (ethanedisulfonic acid or hydrochloride acid) at 1:1 M ratio to the drug. A mixture of dichloromethane (DCM) and methanol (MeOH) (1:1 v/v) was used for DLM free base and DLM chloride ASDs while MeOH was replaced with acetone for DLM edisylate ASDs to eliminate the esterification of the sulfonic acid with the alcohol.

ASDs of DLM free base, DLM chloride, and DLM edisylate were prepared by spray drying using a Buchi Mini Spray Dryer B-290 equipped with an Inert Loop B-295 (Buchi, New Castle, DE). The spray drying process used a feed rate of 4 mL/min, inlet temperature of 75 °C, nitrogen stream flow rate of 700 L/h and aspiration of 35 m^3^/h. ASDs (particle size < 20 µm) were kept in a vacuum oven overnight to remove residual solvents.

#### 2.2.2 Preparation of ASD tablets

The tablet compositions of DLM and DLM salt ASDs with HPMCP are summarized in Table 1. Tablets were compressed using a rotary tablet press, Piccola PLC B (Specialty Measurement Inc, Lebanon, NJ), using #0.4375 size die (Ø 11 mm). Tablets had a hardness of 14–15 kN (measured in a VK 200 Tablet Hardness Tester) and a friability ∼ 0.4% (evaluated using a Vankel Friability Tester, model Friabilator 10800) (Varian Inc., Palo Alto, CA, USA) as measured according to the United States Pharmacopeia, General Chapter <1217> Tablet Breaking Force (USP45-NF40, 2022b) and <1216> Tablet Friability (USP45-NF40, 2022a), respectively.

**Table 1:** Formulation of DLM ASD tablets.

**Core tablet composition**	**Amount (mg)**
ASDs of DLM free base or DLM salt (25% DL)	Equiv. 50 mg DLM
Sodium starch glycolate	40
Croscarmellose sodium	40
Silica, colloidal anhydrous	6
Magnesium stearate	6
Microcrystalline cellulose pH 101	q.s. 500
**Enteric coating layer**	
Acryl EZE® II	75
Deionized water (removed after coating)	750

Tablets were coated with Acryl-EZE® II (composition noted in Table S1) using a Freund Vector coater (Marion, IA). The enteric coating suspension was made by adding 100 g Acryl-EZE® II to 1000 mL water and then stirring for 1 h before coating. The coating parameters included an inlet temperature of 70 °C, bed temperature of 55 °C, coater airflow of 60 CFM, pan rotation speed of 7 rpm, feeding rate of 10 mL/min, and batch size of 500 g (100 g ASD tablets and 400 g placebo tablets of the same size and hardness). After coating, secondary drying was continued for 30 min, using an inlet temperature of 60 °C, coater airflow of 60 CFM, pan rotation speed of 5 rpm. Tablets were cooled to room temperature before packaging. Deltyba® tablets (composition presented in Table S2) were also coated using the same conditions at a batch size of 50 g Deltyba® tablets and 450 g placebo tablets. The weight gain of the enteric coating layer was in the range of 14–16% for all formulations. Tablets with or without enteric coating were packaged and stored in 60 mL HDPE bottles with desiccant at ambient room temperature for further evaluations.

#### 2.2.3 Surface crystallization characterization

Drug crystallization on the ASD surface upon immersion in simulated gastric fluids was detected by polarized light microscopy (PLM) and scanning electron microscopy (SEM). In order to further study surface crystallization, ASD films were prepared by adding 100 µL of an organic solvent solution (see above) of drug, counterion and polymer (10% w/v solid content) to a 22 × 22 mm cover slip. Samples were dried during spinning using a KW-4A spin-coater (Chemat Technology Inc., Northridge, CA) at a speed of 1000 rpm for the first 10 s, followed by 3000 rpm for 45 s under dry air conditions. Samples were kept in a vacuum oven overnight to remove residual solvents. To evaluate the influence of an enteric coating on drug crystallization, a second coating of Eudragit® L 100-55 in MeOH (10% w/v) was applied. 100 µL of a methanolic solution of polymer was introduced and dried rapidly by spinning using the same parameters as for the ASD layer. Residual solvent was removed by storing samples overnight under vacuum. The thickness of each layer was measured by confocal microscopy using a Nikon A1 Confocal and Eclipse Ti2 Inverted Microscope equipped with an Apo 60 × oil λS DIC N2 (numerical aperture 1.4) objective lens (Nikon, Tokyo, Japan). Alexa Fluor 488 (0.001% w/w) and Nile red (0.01% w/w) were added to stock solutions of ASD and Eudragit®, respectively. The uncoated and coated areas on the films were mapped by collecting fluorescent intensity at 488 and 561 nm laser lines for green and red fluorescence.

After immersion in acidic solution (phosphate buffer pH 3.0, composition as described in Table 2), the surface crystallization on ASD films with or without an enteric coating layer was examined using a Nikon Eclipse E600 polarizing microscope (20 × objective) coupled with a Nikon DS-Ri2 camera (Melville, NY). For scanning electron microscopy (SEM), a cover slip coated with the ASD film was mounted on an aluminum stub and coated with platinum using a sputter coater (Cressington Sputter Coater, Watford, UK). SEM images were obtained using a Nova nanoSEM field emission scanning electron microscope (FEI Company, Hillsboro, OR) equipped with an Everhart-Thornley detector at spot size of 3 nm, beam energy of 5 kV and working distance of approximately 5 mm.

**Table 2:** Composition of buffer solutions.

**Composition**	**Gastric fluid**			**Intestinal fluid**	
	**pH 1.6**	**pH 3.0**	**pH 5.0**	**pH 5.8**	**pH 6.5**
Hydrochloric acid (mM)	25.1				
Ortho-phosphoric acid (mM)		5.5			
Monobasic sodium phosphate (mM)		32	133.8		28.4
Sodium hydroxide (mM)			3.1	82	8.7
Maleic acid (mM)				55	
pH (adjusted by HCl 0.1 N or NaOH 0.1 N)	1.6	3.0	5.0	5.8	6.5

#### 2.2.4 Evaluation of gastric resistance of enteric-coated tablets

The gastric resistance of the applied enteric coating was assessed using enteric-coated tablets containing the DLM edisylate ASD. Tablets were incubated overnight in 500 mL of simulated gastric fluids of various pH values at 37 °C under stirring at 150 rpm in a USP apparatus II, Hanson Dissolution System (Billerica, MA). Experiments were conducted in different gastric fluids, including HCl solution pH 1.6; phosphate buffer pH 3.0 and 5.0 (compositions in Table 2); acetate buffer pH 3.0, 4.5 and 6.0 (compositions in Table 3); and high fat simulated fed state gastric media (FEDGAS, pH 3.0, 4.5 and 6.0). The biorelevant medium was prepared by adding FEDGAS gel into acetate buffer solution and stirring for 2 h to obtain a milky solution. All biorelevant media were used within 48 h of preparation according to manufacturer’s guidance (Biorelevant, 2022b).

**Table 3:** Components in homogenized fat dispersion in FEDGAS.

**Composition**	**pH 3.0**	**pH 4.5**	**pH 6.0**
FEDGAS gel (g), including (Biorelevant, 2022a, Leigh et al., 2020):	170	170	170
*Total fat (g)*	*63.8*		
*Bile salts (g)*	*0.34*		
*Stabilizers (g)*	*1.53*		
*Total carbohydrates (g)*	*67.8*		
Sodium citrate dihydrate (g) (Leigh et al., 2020)	0.54	4.46	6.74
Sodium chloride (g) (Leigh et al., 2020)	0.77		
Citric acid (g) (Leigh et al., 2020)	4.45	3.55	0.94
Water (mL)	q.s. 1000 mL	q.s. 1000 mL	q.s. 1000 mL
Average particle size (nm) (Leigh et al., 2020)	160	160	160
Appearance	Milky white color	Milky white color	Milky white color
Buffer capacity (mM/L/△pH) (Biorelevant, 2022b)	22	24	26
Surface tension (mN/m) (Leigh et al., 2020)	40.6	41.3	39.3
Osmolarity (mOsm/L) (Biorelevant, 2022b)	450	460	520

#### 2.2.5 Drug release

Release testing was conducted using a USP apparatus II, Hanson Dissolution System (Billerica, MA) at 37 °C, 150 rpm. The drug release in buffer solutions; FaSSIF V1 and FeSSIF V2 was monitored *in situ* using a Rainbow fiber optic ultraviolet spectrometer coupled with 10-mm pathlength fiber optic probes (Pion Inc, Billerica, MA). The area under the curve over the range of 330–350 nm of the second derivative UV absorbance spectra was used to calculate drug concentration based on a standard curve (drug concentration range of 1–100 µg/mL) obtained in the same dissolution medium. The drug concentration in FEDGAS was measured by high performance liquid chromatography (HPLC) (Agilent Technologies, Santa Clara, CA). Samples were withdrawn at various time intervals, filtered via a 0.2 μm nylon membrane (Pall Corporation, Puerto Rico) and diluted in MeOH before injection of 20 μL solution into the HPLC system. Experiments were run with a C18 column (4.6 × 250 mm, 5 μm), a mobile phase of acetonitrile–water (75–25 v/v) at a flow rate of 1.5 mL/min and UV detection at 320 nm. A calibration curve was built over the drug concentration range of 0.01–50 µg/mL.

Single stage dissolution was conducted in 500 mL intestinal fluids, including phosphate buffer pH 6.5; maleate buffer pH 5.8; FaSSIF V1 and FeSSIF V2. The impact of gastric pH on drug release was evaluated in pH-shift experiments with the first dissolution stage in the gastric compartment for 60 min followed by dissolution at an intestinal pH of 6.5 for an additional 30 min. For the reference formulation (Deltyba®, Otsuka, Japan) and uncoated tablets of DLM ASDs, the volume of gastric medium was 450 mL for HCl solution pH 1.6, 470 mL for phosphate buffer pH 3.0 and 480 mL for phosphate pH 5.0. After the first 60 min, the pH of the solution was adjusted to pH 6.5 by adding 50; 30 and 20 mL, respectively, of 0.57 M phosphate buffer, pH 7.3. The final volume was 500 mL and for complete release, the target drug concentration was 100 µg/mL. The pH measured at the end of experiment fell within the range of 6.4–6.6. For enteric-coated tablets, the gastric medium was discarded after 60 min and the tablet was transferred to intestinal medium and dissolution testing was continued for an additional 60 min.

## 3 Results

### 3.1 Enteric coating to prevent drug crystallization

**Fig. 1:**
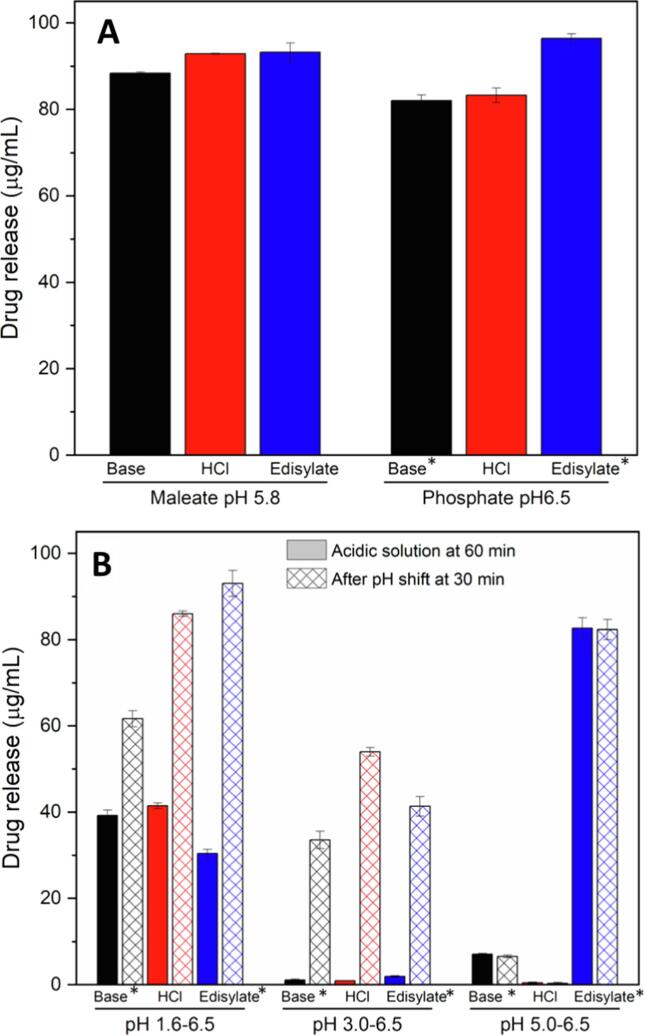
Drug release from tablets containing DLM free base ASD (black); DLM chloride salt ASD (red); and DLM edisylate salt ASD (blue) (25% DL ASDs) in (A) single stage at 60 min or (B) pH-shift dissolution indicating the drug concentration in the gastric medium at 60 min and in the higher pH medium after pH shift and testing for an additional 30 min. Target drug concentration for complete release was 100 µg/mL. *Data taken from a previous study (Nguyen et al., 2023b).

Drug crystallization in ASDs exposed to aqueous solution has been previously identified as a failure mechanism for drug release (Elkhabaz et al., 2019, Jara et al., 2021, Moseson et al., 2022c, Nguyen et al., 2023b, Schittny et al., 2020). Our previous study identified that DLM ASDs with HPMCP underwent crystallization after incubation at certain pH values, with a subsequent impact on release. A subsection of this previously published data, supplemented by additional release studies is summarized in Fig. 1 to provide reference release data for uncoated tablets. Briefly, all formulations showed good release for single-stage testing at higher pH values where HPMCP is soluble, but diminished release for two-stage dissolution tests.

**Fig. 2:**
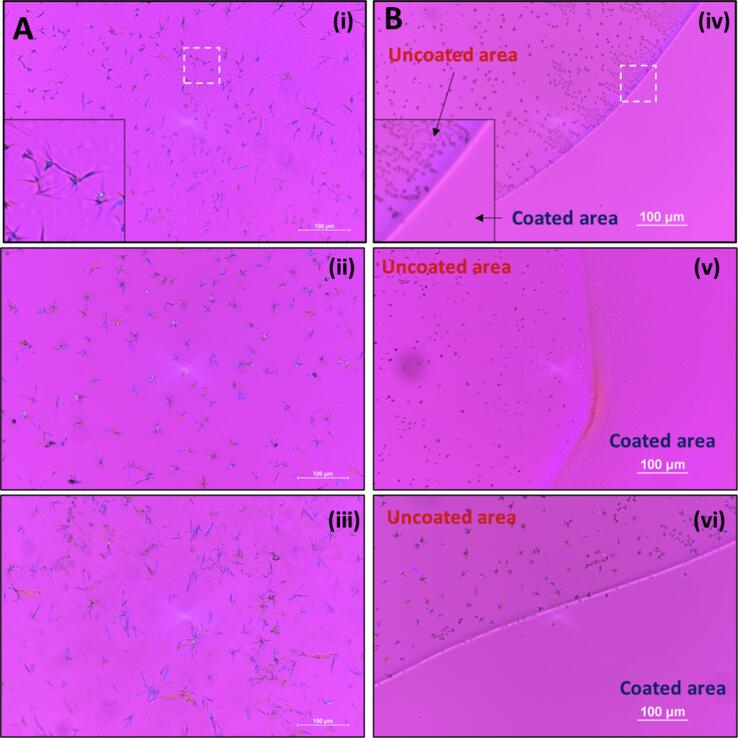
Drug crystallization observed in PLM images of ASD films, including (i; iv) DLM free base ASD; (ii; v) DLM chloride ASD and (iii; vi) DLM edisylate ASD, after immersion in phosphate buffer pH 3.0 for 1 h. (A) Original ASD films. (B) ASD films with enteric coating covering part of the films.

**Fig. 3:**
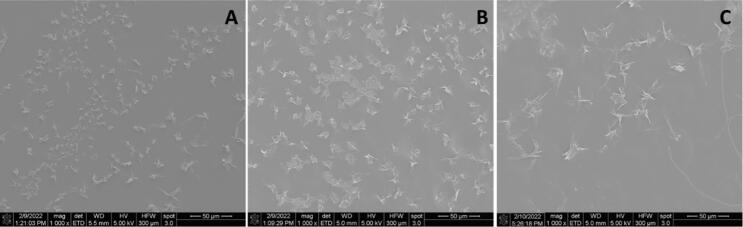
Crystallization observed by SEM in ASD films of (A) DLM free base; (B) DLM edisylate and (C) DLM chloride after immersion in phosphate pH 3.0 for 1 h.

Consistent with the reduced drug release in pH-shift dissolution (Fig. 1B), rapid crystallization was detected on ASD films immersed in phosphate buffer pH 3.0, as demonstrated by PLM images in Fig. 2A and SEM images in Fig. 3. Notably, salt formation did not affect the crystallization tendency of DLM at this pH, where similar outcomes were noted for DLM free base (Fig. 2Ai) and DLM salts (Fig. 2Aii, iii), in agreement with a previous study (Nguyen et al., 2023b).

To evaluate the impact of enteric coating on drug crystallization, a layer of Eudragit® L 100-55 was applied on top of the ASD films. The thickness of the ASD film and enteric coating layer (confirmed by confocal images, Fig. S1) was around 8 and 2 µm, respectively. While crystallization was observed on uncoated areas, the addition of a thin enteric coating layer prevented the formation of crystals following immersion at pH 3.0 (Fig. 2B). Indeed, no drug crystallization was observed even after overnight incubation in gastric solution (Fig. S2).

### 3.2 Gastric resistance of enteric coating

Gastric resistance of enteric coating was evaluated for coated tablets containing the DLM edisylate ASD. For the pH-shift experiments described above, release testing in the gastric compartment was generally evaluated for 30–60 min, reflecting the gastric emptying time in the fasted state (15–60 min) (Grimm et al., 2018, Mudie et al., 2014). However, in the presence of food, the gastric emptying time changes and is within the range of 2–5 h, leading to a prolonged gastric residence time (Al-Gousous et al., 2017, Chen et al., 2008, Dressman et al., 1990, Kalantzi et al., 2006). In addition, food leads to variable gastric pH values (Pavloff et al., 2018). Thus, it is important to evaluate the impact of food, pH and a prolonged residence time on the integrity of an enteric coating.

**Fig. 4:**
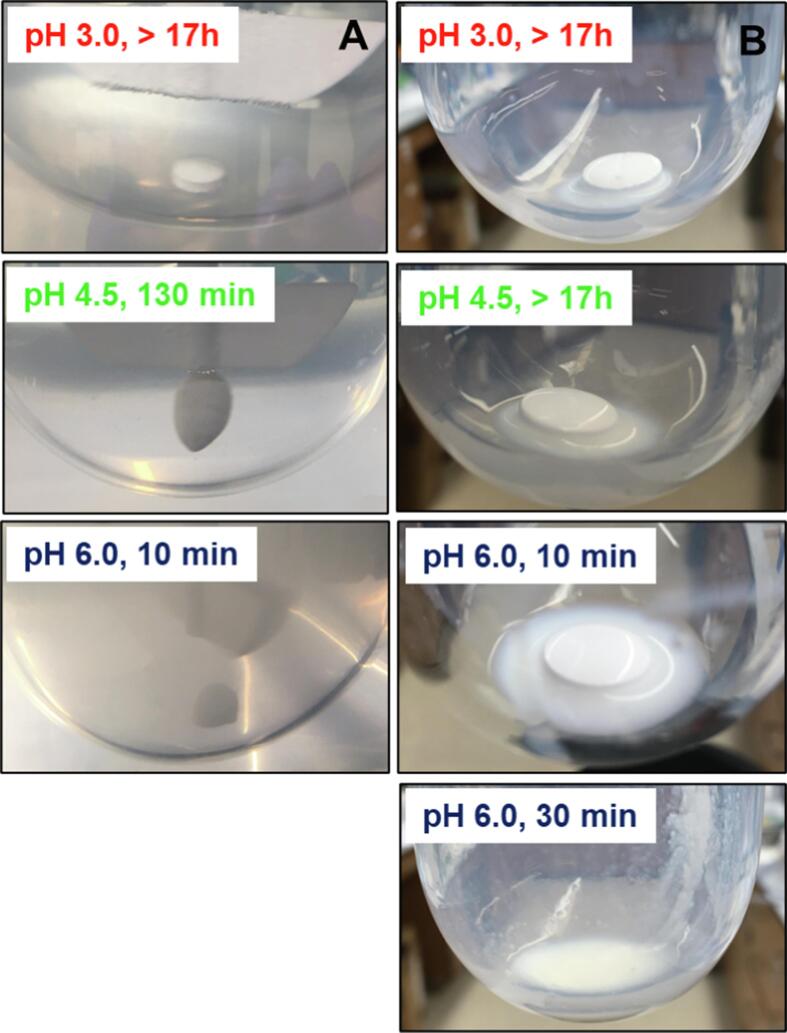
Impact of medium composition and pH on enteric-coated tablet integrity for tablets containing the DLM edisylate ASD (25% DL ASD; 15% enteric coating weight gain) in gastric fluids: (A) Acetate buffer solutions; (B) simulated high-fat gastric media (FEDGAS).

At low pH conditions, the enteric coating prevented tablet disintegration during immersion in gastric buffer solution (acetate buffer pH 3.0) or high-fat simulated medium (FEDGAS 3.0) for more than 17 h (Fig. 4A-B). Similarly, enteric-coated ASD tablets remained intact in HCl solution pH 1.6 or in phosphate buffer pH 3.0 for 15 h, where minimal release was observed (Fig. S3). Enteric-coated tablets passed the general requirements for the tolerance test in gastric solution at higher pH (4.5–5) for 2 h (USP45-NF40, 2022c) with < 0.2% drug release (Table S3). However, in acetate buffer pH 4.5 (Fig. 4A), enteric-coated tablets remained intact for only around ∼ 130 min; and in phosphate buffer pH 5.0 (Fig. S3), failure was observed at about ∼ 150 min. The loss of integrity of the enteric coating allows contact of the ASD with the aqueous medium, resulting in drug leaching (< 5 µg/mL, Table S3) and/or crystallization and notably reduced drug release upon transition to a higher pH medium (Fig. S3). At pH 6.0, the enteric coating layer dissolved within 10–15 min in acetate buffer (Fig. 4A); this represents the gastric pH 30 min post-prandial where the pH range is expected to be 6–6.5 (Andreas et al., 2015, Jantratid et al., 2008, Klein et al., 2004). This is consistent with expectations given that this pH is higher than dissolution pH threshold for the enteric coating material, Eudragit® L 100-55 (Colorcon, 2014).

In the high-fat simulated gastric media (FEDGAS), there are high amounts of glycerides, carbohydrates, and bile salts (Table 3), impacting solution properties, including viscosity, osmolarity, surface tension as well as reducing water diffusivity (Radwan et al., 2014, Radwan et al., 2017). Thus, tablet disintegration was delayed in FEDGAS pH 6.0 to about 20–30 min, compared to less than 15 min in buffer solution (Fig. 4). At a lower pH, specifically FEDGAS pH 4.5, the enteric coating layer retained intact for much longer, more than 17 h, compared to around 2 h in buffer solution at the same pH value (Fig. 4).

### 3.3 Drug release of enteric-coated ASD formulations in buffer solutions of varying pH

**Fig. 5:**
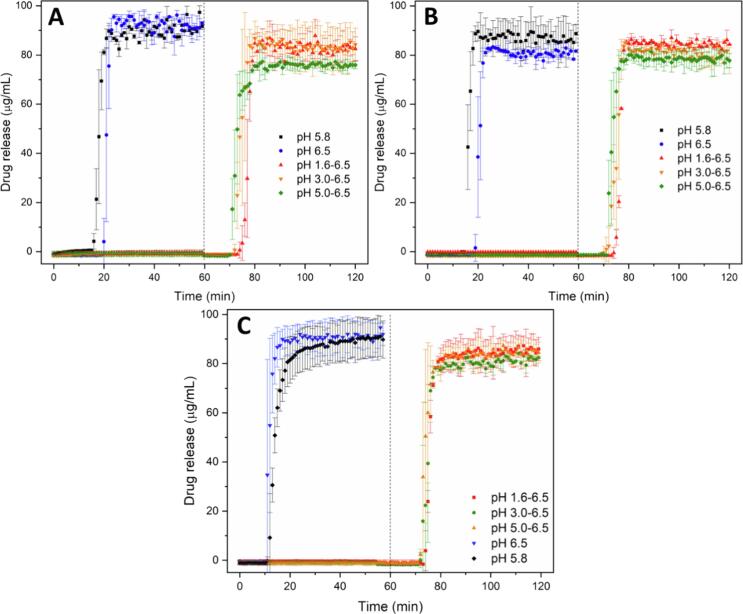
Dissolution profiles of enteric-coated tablets of (A) DLM free base ASD; (B) DLM chloride ASD; (C) DLM edisylate ASD (25% DL ASD) in buffer solutions in single- or two-stage dissolution (dashed line at 60 min indicates pH shift from acidic to high pH medium).

Release testing of enteric-coated tablets of ASDs in buffer solutions was conducted in single stage or pH-shift experiments (Fig. 5). At higher pH condition (> 5.5), a lag time of 10–20 min was observed prior to tablet disintegration and commencement of drug release. A robust drug release was observed for enteric-coated tablets and a supersaturated solution with a concentration of more than 80 µg/mL was generated in single-stage dissolution in maleate buffer pH 5.8, acetate buffer pH 6.0 or phosphate buffer pH 6.5 (Fig. 5 and Fig. S4). For two-stage dissolution, enteric coated tablets showed < 0.5% drug release within 60-min gastric immersion for pH conditions of 5.0 or lower. Following transfer to the simulated intestinal medium (phosphate buffer pH 6.5), release was extensive. Further, variations in the extent of drug release with different simulated gastric pH conditions were much lower for enteric coated tablets, relative to the uncoated formulations (Fig. 1B). Enteric coating provided positive release benefits for both salt ASDs, as well as the free base formulation, with final concentrations of > 80 µg/mL achieved. Enteric coating also minimized differences of drug release from the reference Deltyba® tablets subjected to various pH conditions (Fig. S5), although the overall extent of release was much lower than for the uncoated Deltyba® tablets (Fig. S6).

### 3.4 Drug release in biorelevant media

**Fig. 6:**
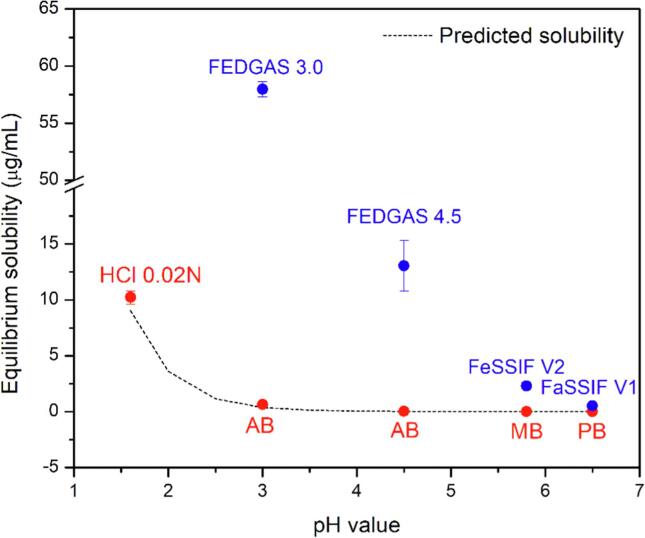
Food component/pH impact on DLM solubility, compared to acetate buffer, maleate buffer and phosphate buffer. The dashed line indicates the equilibrium solubility values predicted by the Henderson-Hasselbalch equation using reported pK_a_ of 4.3 (Shimokawa et al., 2015).

Deltyba® has an improved bioavailability in the fed state (EMA, 2013). Thus, it is of interest to evaluate drug solubility and dissolution of DLM formulation under *in vitro* conditions that capture aspects of different prandial states. The presence of food components showed a remarkable impact on drug solubility (Fig. 6). At pH > 5.5, DLM had very low solubility (< 0.02 µg/mL) in buffer (Nguyen et al., 2023b). With a greater lipid content in FeSSIF V2 (about 7.8 mM) compared to FaSSIF V1 (0.75 mM), the equilibrium solubility in simulated fed state medium was 4.4-fold higher than for fasted state fluid (2.30 ± 0.31 versus 0.52 ± 0.12 µg/mL). Moreover, the solubility in high fat simulated gastric fluids was notably improved, up to 57.97 ± 0.64 and 13.10 ± 2.32 µg/mL for FEDGAS pH 3.0 and pH 4.5, respectively.

**Fig. 7:**
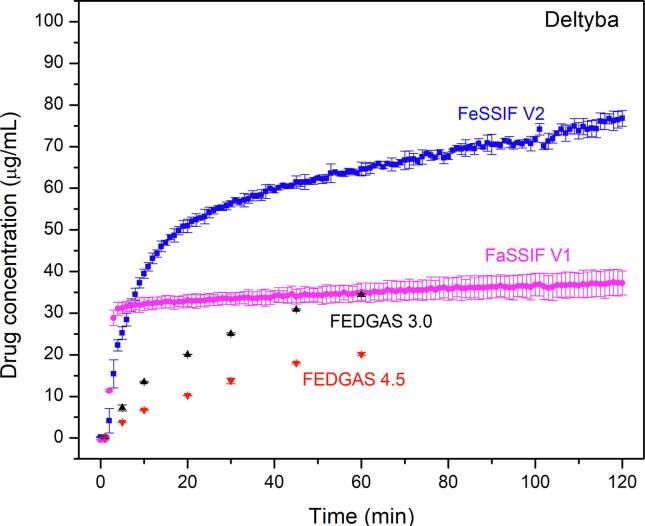
Drug release profiles of Deltyba® tablets in biorelevant media. Drug concentrations in FaSSIF V1 and FeSSIF V2 were monitored *in situ* while release in FEDGAS was measured by HPLC analysis.

The pH- and lipid-dependent solubility contributes to higher drug release from Deltyba® tablets in biorelevant media (Fig. 7). There was an initially rapid and then a more gradual drug release in FeSSIF where the drug concentration reached up to 80 µg/mL after 120 min. This can be compared to a maximum concentration of 35 µg/mL in FaSSIF. In fed stomach conditions, drug release (measured by HPLC) (Fig. 7) was much higher than in the corresponding buffer solution of the same pH value (Fig. S7). pH-shift experiments could not be conducted for FEDGAS systems due to the large amounts of fat and carbohydrate. However, drug crystallization was observed following transfer of Deltyba® to fed-state biorelevant media after initial incubation in a buffer solution representing fed state gastric compartment pH values (Fig. S7).

**Fig. 8:**
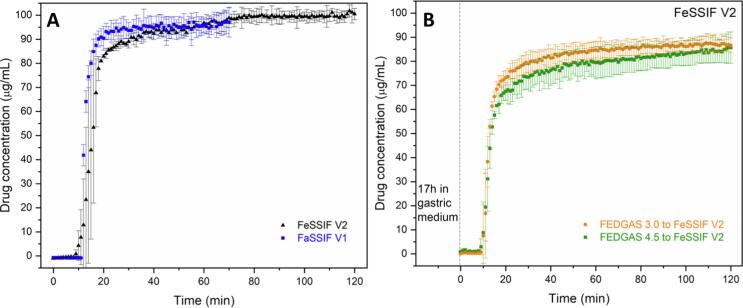
Drug release from enteric-coated tablets of DLM edisylate ASD in (A) simulated intestinal fluids: FaSSIF V1 (blue) and FeSSIF V2 (black); and (B) FeSSIF V2 after 17 h incubation in high-fat simulated gastric fluids. Dashed line indicated pH shift.

Simulated fed-state media had only a minor impact on the release profiles of enteric-coated tablets. Similar release profiles were observed in simulated intestinal media (Fig. 8A) as for buffer solutions of comparable pH (Fig. 5). After a 10–20 min lag time, enteric-coated tablets exhibited near-complete drug release in both fasted and fed-state simulated intestinal media. Following overnight incubation in high-fat gastric fluids, pH 3.0 or 4.5, the extent of drug release following transfer to FeSSIF V2 reached approximately 90 µg/mL (Fig. 8B).

## 4 Discussion

An enteric coating strategy has been applied to ASD formulations in several prior studies (Riekes et al., 2017, Riekes et al., 2016, Smeets et al., 2020). Enteric coating was employed to achieve colonic delivery of a tacrolimus ASD formulated with HPMC, using a coating of Eudragit® L 30 D-55 (Guo et al., 2019). In terms of using coatings to prevent drug crystallization of ASDs, both enteric and non-enteric coatings have been evaluated. ASDs of niclosamide with copovidone (60% DL) exhibited crystallization in a simulated gastric medium (pH 2.0) after immersion for 30 min which could be circumvented by formulating as enteric-coated tablets which delayed drug release until the higher pH environment of the intestine, where crystallization was less favorable due to ionization of the drug (Jara et al., 2022). Non-enteric coatings have also been used to reduce amorphous drug crystallization during storage of the solid formulation (Boel and Van den Mooter, 2023, Li et al., 2019, Zeng et al., 2019). Polymer nanocoating via electrostatic interactions between drug and polymer was applied to improve the stability of amorphous weakly basic drugs, including loratadine (Zeng et al., 2019); clofazimine (Gui et al., 2019, Gui et al., 2021); or a weakly acidic drug, indomethacin (Li et al., 2019). Drug crystallization during storage of high drug loading naproxen-copovidone ASDs was inhibited by applying an additional coating of ethyl cellulose (Boel and Van den Mooter, 2023). More recently, atomic layer coating has attracted attention as an effective surface crystallization inhibitor of high drug loading ASDs of fast crystallizers (Moseson et al., 2022a, Moseson et al., 2022b, Van Duong et al., 2022b). Coatings have been found to reduce surface molecular mobility, notably delaying drug crystallization in the solid state (Gui et al., 2019, Gui et al., 2021, Li et al., 2019, Moseson et al., 2022a, Moseson et al., 2022b, Zeng et al., 2019).

Despite the many examples described above, the application of an enteric coating to an ASD formulated with an enteric polymer, with the goal of preventing crystallization and/or drug release at low pH conditions reflecting the gastric compartment, has not been explored to date. This may be because it is assumed that ASDs formulated with enteric polymers will be protected against crystallization and drug release in the gastric environment due to the insolubility of the enteric polymer. However, despite the low polymer acid solubility, several studies have shown that drugs, in particular weakly basic compounds, are able to partially release from ASDs formulated with enteric polymers under low pH conditions (Elkhabaz et al., 2019, Monschke et al., 2021, Nunes et al., 2022, Wang et al., 2021). Thus, even though the polymer is insoluble at low pH, because the drug is molecularly dispersed within the polymer, some drug release is still observed, whereby the extent of release increases with drug loading. This situation is different from when a tablet or particle has a continuous coating of an enteric polymer, where drug release in the gastric compartment is prevented. Indeed, for DLM, enteric coating of the ASD was able to prevent drug release (< 0.5 μg/mL) for pH conditions of 1.6; 3.0 or 5.0 (Fig. 5), whereas uncoated tablets show a much higher extent of drug release (e.g., release from DLM ASD tablets of ∼ 40; ∼1 and ∼ 7 μg/mL at pH 1.6; 3.0 and 5.0, respectively) (Fig. 1B), even though HPMCP is not soluble until pH 5.

In addition to the extent of drug release, another important factor is the effectiveness of the polymer as a crystallization inhibitor at lower pHs. Enteric polymers, in particularly, HPMCAS and HPMCP, have been found to be effective solution crystallization inhibitors at close to neutral pH conditions where the polymers are ionized and soluble (Amponsah-Efah et al., 2020, Deng et al., 2019, Elkhabaz et al., 2019, Nguyen et al., 2023b, Nunes et al., 2022, Schram et al., 2016, Van Duong et al., 2022a). However, they appear to be less effective crystallization inhibitors at lower pH when polymers become insoluble (Schram et al., 2016). In another study using pyrazinamide and hydrochlorothiazide as model drugs, polymers with the same functional groups may einhibit crystallization or accelerate heteronucleation, depending on their hydrophobicity and solubility (Frank et al., 2019). For DLM, a weakly basic (pK_a_ of 4.3) and rapidly crystallizing drug, combining salt formation and ASD with an enteric polymer (HPMCP-50) (chemical structures of drug and polymer are shown in Fig. S8) was found to be a successful approach to stabilize the amorphous form of the drug in the solid state, as well as in solutions corresponding to intestinal pH values where the polymer is fully ionized (Van Duong et al., 2022a). However, immersion in the gastric compartment at low pH values, where the polymer is insoluble led to the rapid formation of drug crystals on the surface of the ASD (Fig. 2, Fig. 3). These observations can be attributed to the enteric polymer being a poor crystallization inhibitor when it is unionized, and/or the drug having a higher tendency to crystallize when exposed to certain pH conditions. This is particularly true for drug present at the surface of the ASD which is more vulnerable to crystallization due to higher molecular mobility. Furthermore, surface crystallization was found to be detrimental to drug release upon transferring from the gastric to the intestinal compartment, presumably due to seeding by the crystals formed in the gastric compartment which are then released from the ASD matrix when the polymer becomes soluble, accounting for the large variations in release extent from DLM ASDs (Fig. 1B) (Nguyen et al., 2023b).

Incidentally, neither changing enteric polymer (different type/grade) (Nguyen et al., 2023b) nor changing from free base to salt (edisylate or chloride) form could completely eliminate the crystallization issue (Fig. 2A). Specifically, the observed release variability of uncoated DLM tablets (both Deltyba®, and the DLM ASDs tablets herein) can be attributed to a complex interplay of the impact of pH and food components on drug (and polymer) solubility, as well as crystallization tendency. Thus, a general trend observed for all non-commercial DLM tablets is that drug release in single-stage dissolution at higher pH conditions where the polymer is readily soluble (pH 5.8 or above) was fairly similar and nearly complete (Fig. 1A), reflecting the rapid release of the drug into solution where the polymer is able to effectively maintain supersaturation. Two-stage release experiments (Fig. 1B and S6) highlight the potential for impaired release with gastric pH variability, with the worse scenario observed for an initial stage of pH 3.0, where both polymer and drug have low solubility (Nguyen et al., 2023b). Given that *in vitro* testing conditions do not typically encompass a wide variety of gastric pH conditions, these observations may be of importance for *in vivo* behavior where gastric pH is known to be a highly variable parameter, in both the fasted and fed states (Chen et al., 2008, Hatton et al., 2015, Mahar et al., 2012).

We demonstrated herein that enteric coating could successfully prevent drug crystallization when DLM ASD formulations exposed to acidic pH conditions. Inhibition was directly observed via microscopy studies (Fig. 2B), and also inferred from release studies conducted in simple media of different pH values (Fig. 5); release performance of enteric-coated tablets was remarkably improved and less variable relative to that observed for uncoated tablets. However, in conjunction with pH variations, which can arise *in vivo* for a number of reasons (age, administration of acid reducing agents *etc*.), another important consideration impacting DLM formulations is food, in particular fat content. The impact of food components on the integrity of an enteric coating is another relevant consideration. Eudragit® L 100-55 or Eudragit® L 30 D-55 generally resist dissolution at pH values below 5.5 (Rowe et al., 2009). The study of Pavloff and co-workers (Pavloff et al., 2018) on cysteamine bitartrate delayed-release capsules indicated that beads coated with Eudragit® L 30 D-55 showed excellent gastric resistance at pH 5.2 or below. However, foods creating pH values higher than 5.3 were noted to soften the enteric coating and cause premature dissolution of the beads (Pavloff et al., 2018). Herein, an enteric coating of Acryl EZE® II was effective at protecting the tablet core from the gastric environment at pH ≤ 5.0 for at least 2 h in buffer. Interestingly, the presence of food components further slowed down the impairment of the enteric coating layer and prolonged the resistance in simulated gastric fluids (Fig. 4). Drug release upon transfer to intestinal pH medium was found to be the same as for the single stage dissolution if the integrity of enteric coating layer remained intact (Fig. 5). Reduced variability of dissolution was also noted for enteric-coated Deltyba® tablets, albeit at a much lower overall release extent (Fig. S5) relative to the in-house prepared ASD tablets. Once any cracks in the coating occurred, water and buffer species could penetrate into the core, resulting in drug crystallization and decreased drug release (Fig. S3). Furthermore, the presence of food components showed only a minor impact on *in vitro* performance of enteric-coated ASD tablets. With enteric coating, ASD tablets resulted in near complete drug release either in both buffer solutions or in fasted/fed simulated media (Figs. 5 and 8). Thus, this protective coating layer appears to be a useful potential strategy to reduce the release variability due to the large pH range in the stomach between the fasted and fed states.

It is also important to consider the crystallization tendency of the ASD formulations and the potential impact on absorption in the context of drug solubility in different media (Van Duong et al., 2022c). Clearly, crystallization leads to very poor overall DLM release in buffer due to the low equilibrium solubility of the crystalline form. However, drug equilibrium solubility in fed-state simulated gastric fluid (pH 4.5) and fed-state simulated intestinal media was approximately 650- and 100-fold higher, respectively, than in corresponding buffer solutions (Fig. 6). The higher solubility likely accounts for the remarkably improved drug release from Deltyba® in the presence of components that model food and bile salts (Fig. 7), compared to in buffer (Figs. S6 and S7) during single-stage testing. In other words, the impact of any crystallization is mitigated by the higher solubility in these media. Further, the food-dependent solubility and dissolution likely contribute to the positive food impact on bioavailability of poorly soluble compounds like posaconazole (Krishna et al., 2012), ziprasidone (Xue et al., 2019) or pretomanid (Nguyen et al., 2023a). The improved exposure in fed state was also observed for Deltyba® in clinical studies, but also to the high variability observed between different subjects (EMA, 2013). Studies revealed that administration of a 200 mg dose of Deltyba® with a standard meal led to much higher C*_max_* and AUC*_0-inf_* values (increases of 3.4- and 2.9-fold, respectively), versus fasted conditions (EMA, 2013). Moreover, the bioavailability of DLM was found to vary depending on dietary fat intake. For a single dose of a 400 mg Deltyba® tablet, the mean C_max_ increased by 327% and 213%, and the AUC*_0-inf_* increased by 347% and 206%, after a high-fat meal or a standard meal, respectively, compared to fasting conditions (EMA, 2013). Additional support for the importance of fat for DLM absorption comes from a population pharmacokinetic study of Deltyba® in patients with multidrug-resistant tuberculosis (MDR-TB), where medication was always taken with food. It was noted that there were differences in relative bioavailability and absorption rate between evening and morning doses, which were postulated to result from differences in the fat content of the food ingested around the time of dosing (Wang et al., 2020). Given that many of the patients affected by MDR-TB may not have access to high fat meals, our observations that the release of DLM from ASDs can be both improved in the absence of solubilizing components and rendered less variable as a function of gastric pH by using an enteric coating strategy, may be of practical importance.

## 5 Conclusions

ASD formulations of delamanid with an enteric polymer showed compromised release following immersion in gastric pH conditions prior to transfer to intestinal pH conditions, attributed to surface drug crystallization. The deleterious release behavior could be remediated via application of an enteric coating to the ASD tablet, thereby preventing drug surface crystallization under acidic conditions. This strategy may represent an approach to reduce *in vivo* absorption variability arising from different pH and media conditions associated with prandial state.

## CRediT authorship contribution statement

**Hanh Thuy Nguyen:** Conceptualization, Methodology, Investigation, Writing – original draft, Formal analysis, Visualization. **Tu Van Duong:** Conceptualization, Methodology, Investigation, Writing – review & editing, Formal analysis, Visualization. **Lynne S. Taylor:** Conceptualization, Writing – review & editing, Supervision, Funding acquisition.

## Declaration of Competing Interest

The authors declare that they have no known competing financial interests or personal relationships that could have appeared to influence the work reported in this paper.

## Data Availability

Data will be made available on request.

## References

[b0005] Al-Gousous J., Tsume Y., Fu M., Salem I.I., Langguth P. (2017). Unpredictable performance of pH-dependent coatings accentuates the need for improved predictive in vitro test systems. Mol. Pharm..

[b0010] Amponsah-Efah K.K., Mistry P., Eisenhart R., Suryanarayanan R. (2020). The influence of the strength of drug–polymer interactions on the dissolution of amorphous solid dispersions. Mol. Pharm..

[b0015] Andreas C.J., Chen Y.-C., Markopoulos C., Reppas C., Dressman J. (2015). In vitro biorelevant models for evaluating modified release mesalamine products to forecast the effect of formulation and meal intake on drug release. Eur. J. Pharm. Biopharm..

[b0020] Bhugra C., Pikal M.J. (2008). Role of thermodynamic, molecular, and kinetic factors in crystallization from the amorphous state. J. Pharm. Sci..

[b0025] Biorelevant, 2022a. Certificate of Analysis: FEDGAS Gel.

[b0030] Biorelevant, 2022b. Technical Data Sheet for FEDGAS Discriminatory Dissolution Media.

[b0035] Boel E., Van den Mooter G. (2023). The impact of applying an additional polymer coating on high drug-loaded amorphous solid dispersions layered onto pellets. Int. J. Pharm..

[b0040] Chen E.P., Mahar Doan K.M., Portelli S., Coatney R., Vaden V., Shi W. (2008). Gastric pH and gastric residence time in fasted and fed conscious cynomolgus monkeys using the Bravo® pH system. Pharm. Res..

[b0045] Colorcon, 2014. Product lnformation on Acryl-EZE® II Optimized Aqueous Acrylic Enteric System. Colorcon.

[b0050] Deng Y., Liang Q., Wang Y., Zhang X., Yan C., He Y. (2019). The inhibiting role of hydroxypropylmethylcellulose acetate succinate on piperine crystallization to enhance its dissolution from its amorphous solid dispersion and permeability. RSC Adv..

[b0055] Dressman J.B., Berardi R.R., Dermentzoglou L.C., Russell T.L., Schmaltz S.P., Barnett J.L., Jarvenpaa K.M. (1990). Upper gastrointestinal (GI) pH in young, healthy men and women. Pharm. Res..

[b0060] Elkhabaz A., Sarkar S., Simpson G.J., Taylor L.S. (2019). Characterization of phase transformations for amorphous solid dispersions of a weakly basic drug upon dissolution in biorelevant media. Pharm. Res..

[b0065] EMA (2013).

[b0070] Evonik, 2020. Technical Information Eudragit L 100-55. EIP/Product Regulatory Datasheet. Evonik Industries, CRS.

[b0075] Frank D.S., Zhu Q., Matzger A.J. (2019). Inhibiting or accelerating crystallization of pharmaceuticals by manipulating polymer solubility. Mol. Pharm..

[b0080] Friesen D.T., Shanker R., Crew M., Smithey D.T., Curatolo W., Nightingale J. (2008). Hydroxypropyl methylcellulose acetate succinate-based spray-dried dispersions: an overview. Mol. Pharm..

[b0085] Gan K.H., Geus W., Bakker W., Lamers C., Heijerman H. (1996). In vitro dissolution profiles of enteric-coated microsphere/microtablet pancreatin preparations at different pH values. Aliment. Pharmacol. Ther..

[b0090] Grimm M., Koziolek M., Kühn J.-P., Weitschies W. (2018). Interindividual and intraindividual variability of fasted state gastric fluid volume and gastric emptying of water. Eur. J. Pharm. Biopharm..

[b0095] Gui Y., Chen Y., Chen Z., Jones K.J., Yu L. (2019). Improving stability and dissolution of amorphous clofazimine by polymer nano-coating. Pharm. Res..

[b0100] Gui Y., McCann E.C., Yao X., Li Y., Jones K.J., Yu L. (2021). Amorphous drug-polymer salt with high stability under tropical conditions and fast dissolution: the case of clofazimine and poly(acrylic acid). Mol. Pharm..

[b0105] Guo J., Fang H., Gui S., Huang Y. (2019). Solid dispersion-based pellet for colon delivery of tacrolimus through time-and pH-dependent layer coating: preparation, in vitro and in vivo studies. Braz. JPharm. Sci..

[b0110] Hatton G.B., Yadav V., Basit A.W., Merchant H.A. (2015). Animal farm: considerations in animal gastrointestinal physiology and relevance to drug delivery in humans. J. Pharm. Sci.

[b0115] Hiew, T.N., Saboo, S., Zemlyanov, D.Y., Punia, A., Wang, M., Smith, D., Lowinger, M., Solomos, M.A., Schenck, L., Taylor, L.S., 2022a. Improving dissolution performance and drug loading of amorphous dispersions through a hierarchical particle approach. J. Pharm. Sci. 10.1016/j.xphs.2022.12.019 36574837

[b0120] Hiew T.N., Zemlyanov D.Y., Taylor L.S. (2022). Balancing solid-state stability and dissolution performance of lumefantrine amorphous solid dispersions: the role of polymer choice and drug-polymer interactions. Mol. Pharm..

[b0125] Jantratid E., Janssen N., Reppas C., Dressman J.B. (2008). Dissolution media simulating conditions in the proximal human gastrointestinal tract: an update. Pharm. Res..

[b0130] Jara M.O., Warnken Z.N., Williams R.O. (2021). Amorphous solid dispersions and the contribution of nanoparticles to in vitro dissolution and in vivo testing: niclosamide as a case study. Pharmaceutics.

[b0135] Jara M.O., Warnken Z.N., Sahakijpijarn S., Thakkar R., Kulkarni V.R., Christensen D.J., Koleng J.J., Williams R.O. (2022). Oral delivery of niclosamide as an amorphous solid dispersion that generates amorphous nanoparticles during dissolution. Pharmaceutics.

[b0140] Kalantzi L., Goumas K., Kalioras V., Abrahamsson B., Dressman J.B., Reppas C. (2006). Characterization of the human upper gastrointestinal contents under conditions simulating bioavailability/bioequivalence studies. Pharm. Res..

[b0145] Klein S., Dressman J.B., Butler J., Hempenstall J.M., Reppas C. (2004). Media to simulate the postprandial stomach I. Matching the physicochemical characteristics of standard breakfasts. J. Pharm. Pharmacol..

[b0150] Krishna G., Ma L., Martinho M., O'Mara E. (2012). Single-dose phase I study to evaluate the pharmacokinetics of posaconazole in new tablet and capsule formulations relative to oral suspension. Antimicrobial Agents Chemother..

[b0155] Leigh, M.L., Dos Santos, V.R., Leigh, S., 2020. Biorelevant composition. WO patent 2020/201779A1.

[b0160] Li Y., Yu J., Hu S., Chen Z., Sacchetti M., Sun C.C., Yu L. (2019). Polymer nanocoating of amorphous drugs for improving stability, dissolution, powder flow, and tabletability: the case of chitosan-coated indomethacin. Mol. Pharm..

[b0165] Maderuelo C., Lanao J.M., Zarzuelo A. (2019). Enteric coating of oral solid dosage forms as a tool to improve drug bioavailability. Eur. J. Pharm. Sci..

[b0170] Mahar K.M., Portelli S., Coatney R., Chen E.P. (2012). Gastric pH and gastric residence time in fasted and fed conscious beagle dogs using the Bravo® pH system. J. Pharm. Sci.

[b0175] Monschke M., Kayser K., Wagner K.G. (2021). Influence of particle size and drug load on amorphous solid dispersions containing pH-dependent soluble polymers and the weak base ketoconazole. AAPS PharmSciTech.

[b0180] Monschke M., Wagner K.G. (2019). Amorphous solid dispersions of weak bases with pH-dependent soluble polymers to overcome limited bioavailability due to gastric pH variability–an in-vitro approach. Int. J. Pharm..

[b0185] Moseson D.E., Benson E.G., Cao Z., Bhalla S., Wang F., Wang M., Zheng K., Narwankar P.K., Simpson G.J., Taylor L.S. (2022). Impact of aluminum oxide nanocoating on drug release from amorphous solid dispersion particles. Mol. Pharm..

[b0190] Moseson D.E., Benson E.G., Nguyen H.T., Wang F., Wang M., Zheng K., Narwankar P.K., Taylor L.S. (2022). Atomic layer coating to inhibit surface crystallization of amorphous pharmaceutical powders. ACS Appl. Mater. Interfaces.

[b0195] Moseson D.E., Hiew T.N., Su Y., Taylor L.S. (2022). Formulation and processing strategies which underpin susceptibility to matrix crystallization in amorphous solid dispersions. J. Pharm. Sci..

[b0200] Mudie D.M., Murray K., Hoad C.L., Pritchard S.E., Garnett M.C., Amidon G.L., Gowland P.A., Spiller R.C., Amidon G.E., Marciani L. (2014). Quantification of gastrointestinal liquid volumes and distribution following a 240 mL dose of water in the fasted state. Mol. Pharm..

[b0205] Nguyen D.N., Palangetic L., Clasen C., Van den Mooter G. (2016). One-step production of darunavir solid dispersion nanoparticles coated with enteric polymers using electrospraying. J. Pharm. Pharmacol..

[b0210] Nguyen H.T., Van Duong T., Jaw-Tsai S., Bruning-Barry R., Pande P., Taneja R., Taylor L.S. (2023). Fed- and fasted-state performance of pretomanid amorphous solid dispersions formulated with an enteric polymer. Mol. Pharm..

[b0215] Nguyen H.T., Van Duong T., Taylor L.S. (2023). Impact of gastric pH variations on the release of amorphous solid dispersion formulations containing a weakly basic drug and enteric polymers. Mol. Pharm..

[b0220] Nunes P.D., Pinto J.F., Henriques J., Paiva A.M. (2022). Insights into the release mechanisms of ITZ:HPMCAS amorphous solid dispersions: the role of drug-rich colloids. Mol. Pharm..

[b0225] Pavloff N., Hauser T.A., Williams C., Isbell S.L., Cadieux B., Johnson M. (2018). The effect of food and liquid pH on the integrity of enteric-coated beads from cysteamine bitartrate delayed-release capsules. Drug Des. Devel. Ther..

[b0230] Radwan A., Wagner M., Amidon G.L., Langguth P. (2014). Bio-predictive tablet disintegration: effect of water diffusivity, fluid flow, food composition and test conditions. Eur. J. Pharm. Sci..

[b0235] Radwan A., Zaid A.N., Jaradat N., Odeh Y. (2017). Food effect: the combined effect of media pH and viscosity on the gastrointestinal absorption of ciprofloxacin tablet. Eur. J. Pharm. Sci..

[b0240] Riekes M.K., Engelen A., Appeltans B., Rombaut P., Stulzer H.K., Van den Mooter G. (2016). New perspectives for fixed dose combinations of poorly water-soluble compounds: a case study with Ezetimibe and Lovastatin. Pharm. Res..

[b0245] Riekes M.K., Dereymaker A., Berben P., Augustijns P., Stulzer H.K., Van den Mooter G. (2017). Development of enteric-coated fixed dose combinations of amorphous solid dispersions of ezetimibe and lovastatin: Investigation of formulation and process parameters. Int. J. Pharm..

[b0250] Rowe R.C., Sheskey P., Quinn M. (2009).

[b0255] Schittny A., Philipp-Bauer S., Detampel P., Huwyler J., Puchkov M. (2020). Mechanistic insights into effect of surfactants on oral bioavailability of amorphous solid dispersions. J. Control. Release.

[b0260] Schram C.J., Beaudoin S.P., Taylor L.S. (2016). Polymer inhibition of crystal growth by surface poisoning. Cryst. Growth Des..

[b0265] Shimokawa Y., Sasahara K., Koyama N., Kitano K., Shibata M., Yoda N., Umehara K. (2015). Metabolic mechanism of delamanid, a new anti-tuberculosis drug, in human plasma. Drug Metab. Dispos..

[b0270] Siepmann F., Siepmann J., Walther M., MacRae R., Bodmeier R. (2006). Aqueous HPMCAS coatings: effects of formulation and processing parameters on drug release and mass transport mechanisms. Eur. J. Pharm. Biopharm..

[b0275] Smeets A., Re I.L., Clasen C., Van den Mooter G. (2020). Fixed dose combinations for cardiovascular treatment via coaxial electrospraying: coated amorphous solid dispersion particles. Int. J. Pharm..

[b0280] Thoma K., Bechtold K. (1999). Influence of aqueous coatings on the stability of enteric coated pellets and tablets. Eur. J. Pharm. Biopharm..

[b0285] Ting J.M., Navale T.S., Jones S.D., Bates F.S., Reineke T.M. (2015). Deconstructing HPMCAS: excipient design to tailor polymer–drug interactions for oral drug delivery. ACS Biomater. Sci. Eng..

[b0290] Tran, H., Patel, P.J., Aburub, A., Sperry, A., Estwick, S., ElSayed, M.E., –Mannan, A.D., 2022. Identification of a multi-component formulation for intestinal delivery of a GLP-1/glucagon co-agonist peptide. Pharm. Res. 39, 2555–2567. 10.1007/s11095-022-03372-1 36050547

[b0295] Trasi N.S., Oucherif K.A., Litster J.D., Taylor L.S. (2015). Evaluating the influence of polymers on nucleation and growth in supersaturated solutions of acetaminophen. CrstEngComm.

[b0300] Ueda K., Higashi K., Kataoka M., Yamashita S., Yamamoto K., Moribe K. (2014). Inhibition mechanism of hydroxypropyl methylcellulose acetate succinate on drug crystallization in gastrointestinal fluid and drug permeability from a supersaturated solution. Eur. J. Pharm. Sci..

[b0310] USP45-NF40, 2022a. <1216> Tablet Friability. United States Pharmacopeial Convention, Rockville, MD, USA.

[b0315] USP45-NF40, 2022b. <1217> Tablet Breaking Force. The United States Pharmacopeial Convention, Rockville, MD, USA.

[b0305] USP45-NF40, 2022c. <711> Dissolution. The United States Pharmacopeial Convention, Rockville, MD, USA.

[b0320] Van Duong T., Nguyen H.T., Taylor L.S. (2022). Combining enabling formulation strategies to generate supersaturated solutions of delamanid: in situ salt formation during amorphous solid dispersion fabrication for more robust release profiles. JEur. J. Pharm. Biopharm..

[b0325] Van Duong T., Nguyen H.T., Wang F., Wang M., Narwankar P.K., Taylor L.S. (2022). Surface nanocoating of high drug-loading spray-dried amorphous solid dispersions by atomic layer coating: Excellent physical stability under accelerated storage conditions for two years. Int. J. Pharm..

[b0330] Van Duong T., Ni Z., Taylor L.S. (2022). Phase behavior and crystallization kinetics of a poorly water-soluble weakly basic drug as a function of supersaturation and media composition. Mol. Pharm..

[b0335] Wang, X., Mallikaarjun, S., Gibiansky, E., 2020. Population pharmacokinetic analysis of delamanid in patients with pulmonary multidrug-resistant tuberculosis. Antimicrobial Agents Chemother. 65, e01202–01220. 10.1128/AAC.01202-20 PMC7927850 33106258

[b0340] Wang B., Nethercott M.J., Narula A., Hanrahan M., Kuang S., Wenslow R.M., Li N. (2021). pH-Dependent supersaturation from amorphous solid dispersions of weakly basic drugs. Pharm. Res..

[b0345] Xie T., Taylor L.S. (2016). Dissolution performance of high drug loading celecoxib amorphous solid dispersions formulated with polymer combinations. Pharm. Res..

[b0350] Xue X., Chen G., Xu X., Wang J., Wang J., Ren L. (2019). A combined utilization of Plasdone-S630 and HPMCAS-HF in ziprasidone hydrochloride solid dispersion by hot-melt extrusion to enhance the oral bioavailability and no food effect. AAPS PharmSciTech.

[b0355] Zeng A., Yao X., Gui Y., Li Y., Jones K.J., Yu L. (2019). Inhibiting surface crystallization and improving dissolution of amorphous loratadine by dextran sulfate nanocoating. J. Pharm. Sci..

