# Supplementary material for: Enteric coating of tablets containing an amorphous solid dispersion of an enteric polymer and a weakly basic drug: A strategy to enhance in vitro release
Source: Int J Pharm. 2023 Jul 25;642:123139. doi: 10.1016/j.ijpharm.2023.123139 (PMC10390825; doi:10.1016/j.ijpharm.2023.123139)
Supplement: Supplementary data 1 [file mmc1.docx]

**Supplementary information**

**Enteric coating of tablets containing an amorphous solid dispersion of an enteric polymer and a weakly basic drug: a strategy to enhance in vitro release**

Hanh Thuy Nguyen^#^, Tu Van Duong^#^, and Lynne S. Taylor^*^

Department of Industrial and Physical Pharmacy, College of Pharmacy, Purdue University, West Lafayette, Indiana 47907, United States

^*^ Corresponding author. E-mail: lstaylor@purdue.edu. Tel: +1 (765) 496-6614. Fax: +1 (765) 494-6545.

^#^ Nguyen, H.T and Van Duong T. contributed equally to this study.

**Table S1: Composition of Acryl EZE II**^®^ **formulation (Lot #493Z180022) (Colorcon, 2014)**

| **No.** | **Ingredients** |
| --- | --- |
| 1 | Methacrylic acid copolymer |
| 2 | Talc |
| 3 | Titanium dioxide |
| 4 | Poloxamer 407 |
| 5 | Calcium silicate |
| 6 | Sodium bicarbonate |
| 7 | Sodium lauryl sulfate |

**Table S2: Composition of Deltyba**^®^ **tablets (EMA, 2013)**

| **Tablet core** | **Film coating** |
| --- | --- |
| Delamanid | Hypromellose |
| Hypromellose phthalate | Macrogol 8000 |
| Povidone | Titanium dioxide |
| All-rac- α-Tocopherol | Talc |
| Cellulose, microcrystalline | Iron oxide yellow (E127) |
| Sodium starch glycolate (Type A) |  |
| Silica, colloidal hydrated |  |
| Magnesium stearate |  |
| Lactose monohydrate |  |


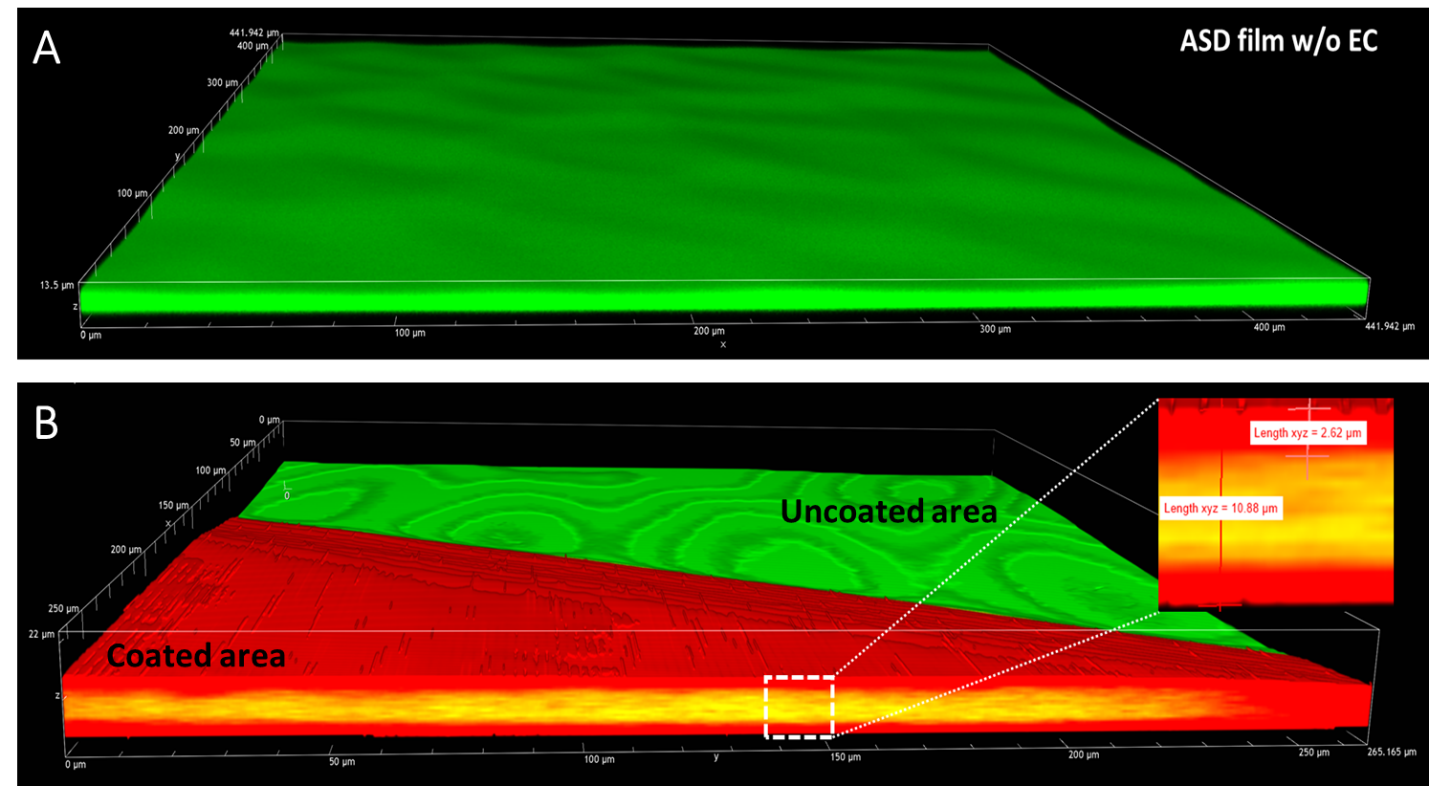


**Figure S1:** Confirmation of enteric coating layer on ASD film of DLM chloride under confocal microscopy. Alexa Fluor 488 (0.001% w/w) and Nile red (0.01% w/w) were added to the ASD and Eudragit^®^ L100-55 solution, respectively.


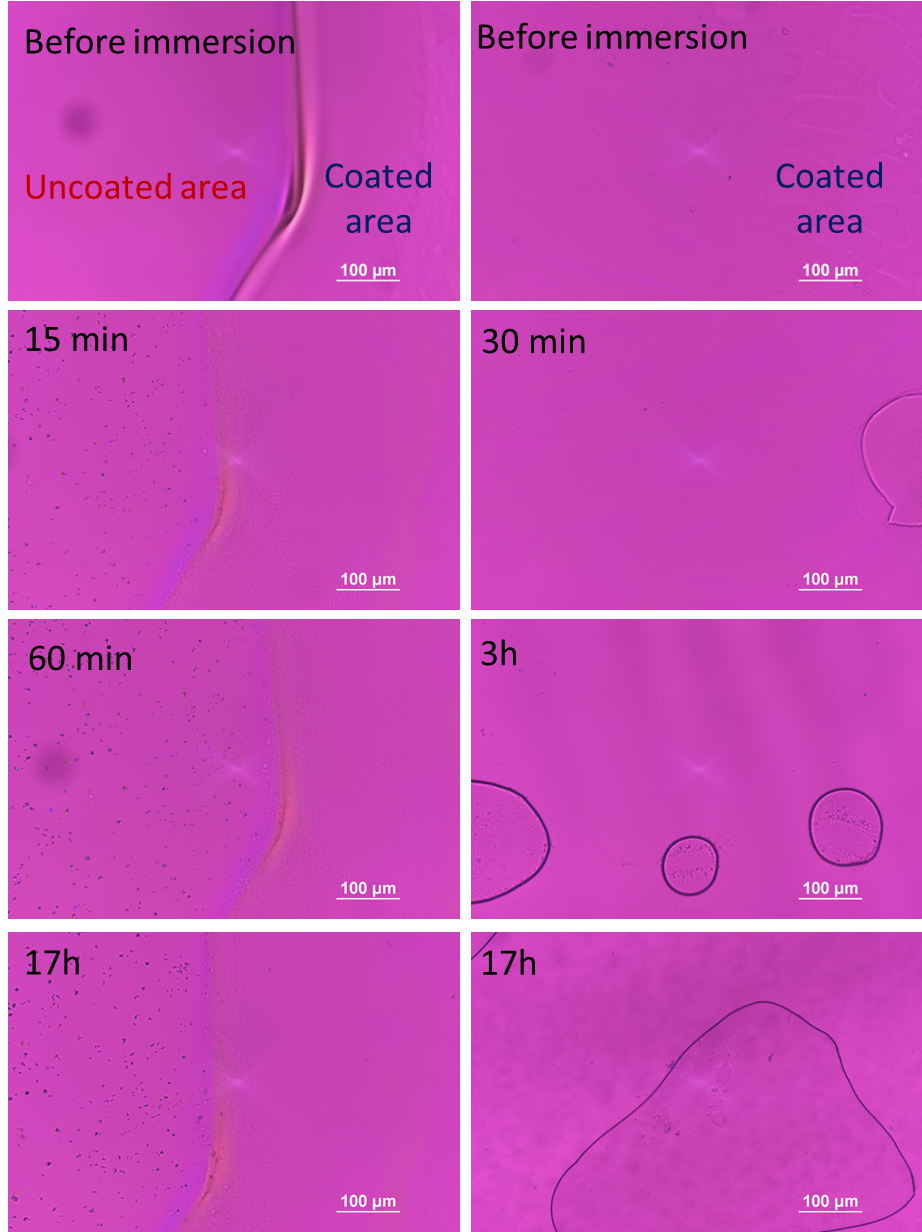


**Figure S2: PLM images showing that no crystallization was observed on an enteric-coated ASD film of DLM chloride after overnight incubation in phosphate buffer pH 3.0.**


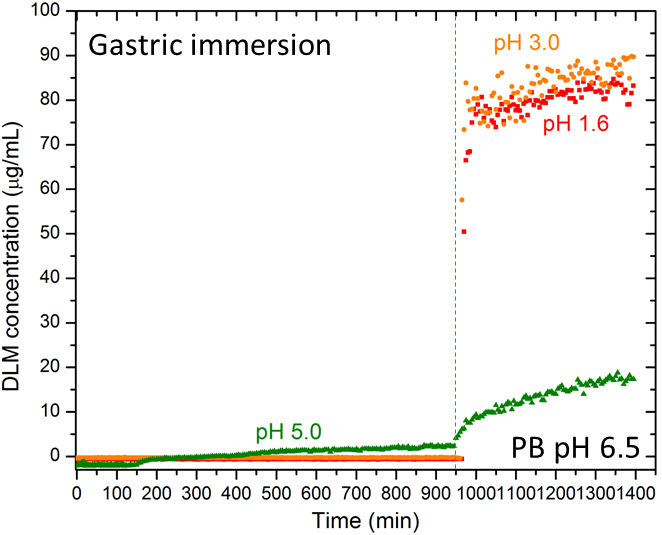


**Figure S3: Release versus time profiles showing the gastric resistance of enteric-coated tablets of DLM edisylate ASD in HCl pH 1.6 or phosphate buffer solution.**

**Table S3: Drug release (%) in different gastric fluids. (AB: Acetate buffer; PB: Phosphate buffer)**

| **Gastric fluid** | **Time (h)** | | | | |
| --- | --- | --- | --- | --- | --- |
|  | **0.5** | **2** | **2.5** | **3** | **16** |
| **Buffer only** |  |  |  |  |  |
| ***HCl pH 1.6*** | <0.2 | <0.2 | <0.2 | <0.2 | <0.2 |
| ***AB pH 3.0*** | <0.2 | <0.2 | <0.2 | <0.2 | <0.2 |
| ***PB pH 3.0*** | <0.2 | <0.2 | <0.2 | <0.2 | <0.2 |
| ***AB pH 4.5*** | <0.2 | <0.2 | <1 | <1 | - |
| ***PB pH 5.0*** | <0.2 | <0.2 | <1 | <2 | <5 |
| ***AB pH 6.0*** | >80 | - | - | - | - |
| **Biorelevant media** |  |  |  |  |  |
| ***FaSSGF pH 1.6*** | <0.2 | <0.2 | <0.2 | <0.2 | <0.2 |
| ***FEDGAS pH 3.0**** | - | - | - | - | <0.2 |
| ***FEDGAS pH 4.5**** | - | - | - | - | <0.2 |
| ***FEDGAS pH 6.0*** | N/A |  |  |  |  |

**Drug concentration was measured by HPLC*

*N/A: Not applicable because filtration could not be applied for supersaturated solution.*





**Figure S4: Drug release of enteric-coated tablets of DLM edisylate ASD in acetate buffer pH 6.0.**





**Figure S5: Dissolution of enteric-coated Deltyba**^®^ **tablets in buffer solutions in single- or two-stage dissolution (dashed line indicates pH shift from acidic to high pH medium).**





**Figure S6: Dissolution variation of Deltyba**^®^ **tablets in different buffer solutions with release profile in phosphate buffer pH 6.5 and pH shift from 1.6 to 6.5 as reported in a previous study (Van Duong et al., 2022).**


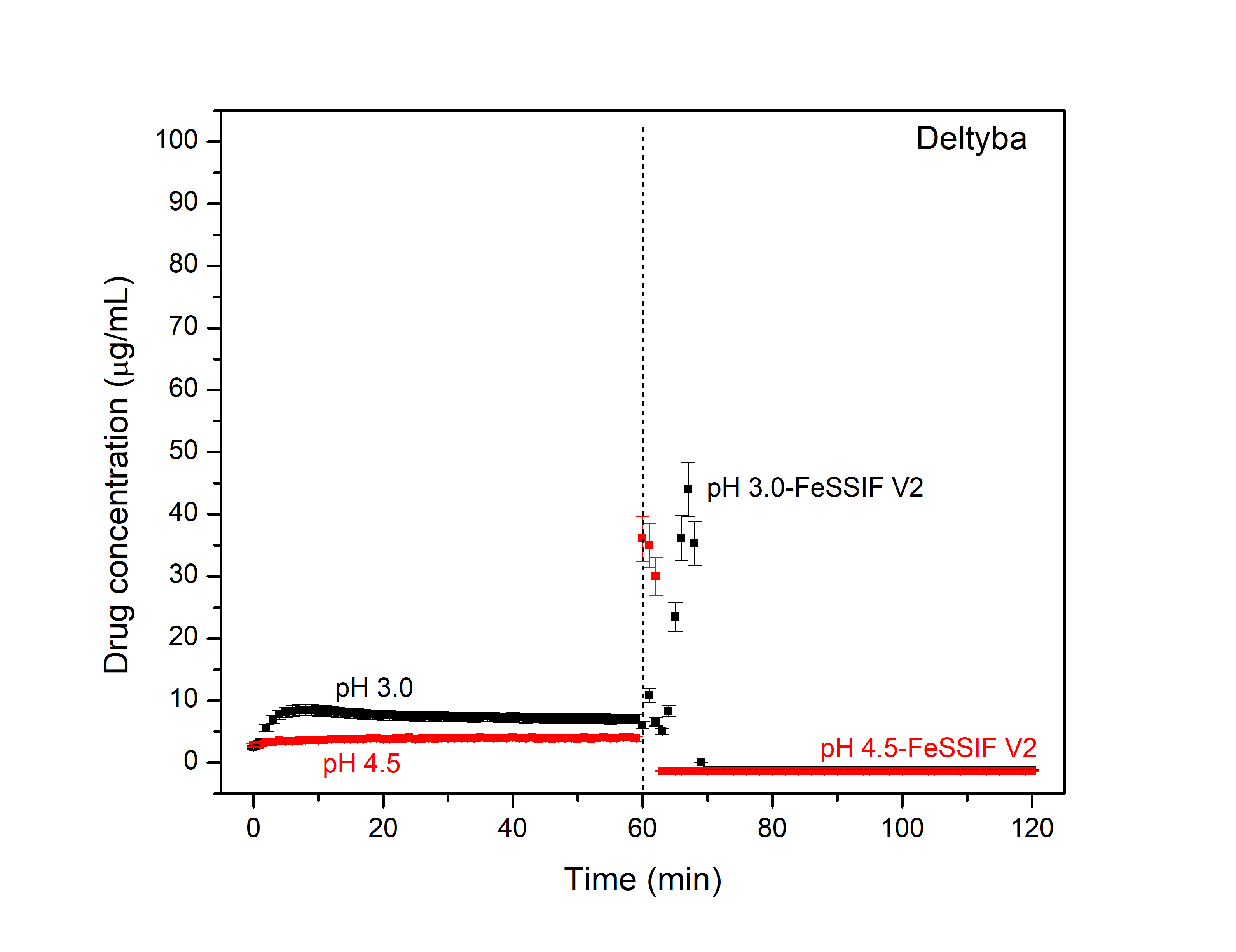


**Figure S7: Dissolution of Deltyba**^®^ **tablets in 450 mL and 470 mL acetate buffer solutions pH 3.0 and 4.5, respectively, followed by transfer to FeSSIF V2, pH 5.8 (by addition of 50 and 30 mL 10x FeSSIF solution, pH 12.6, maleate buffer 0.55 M).**


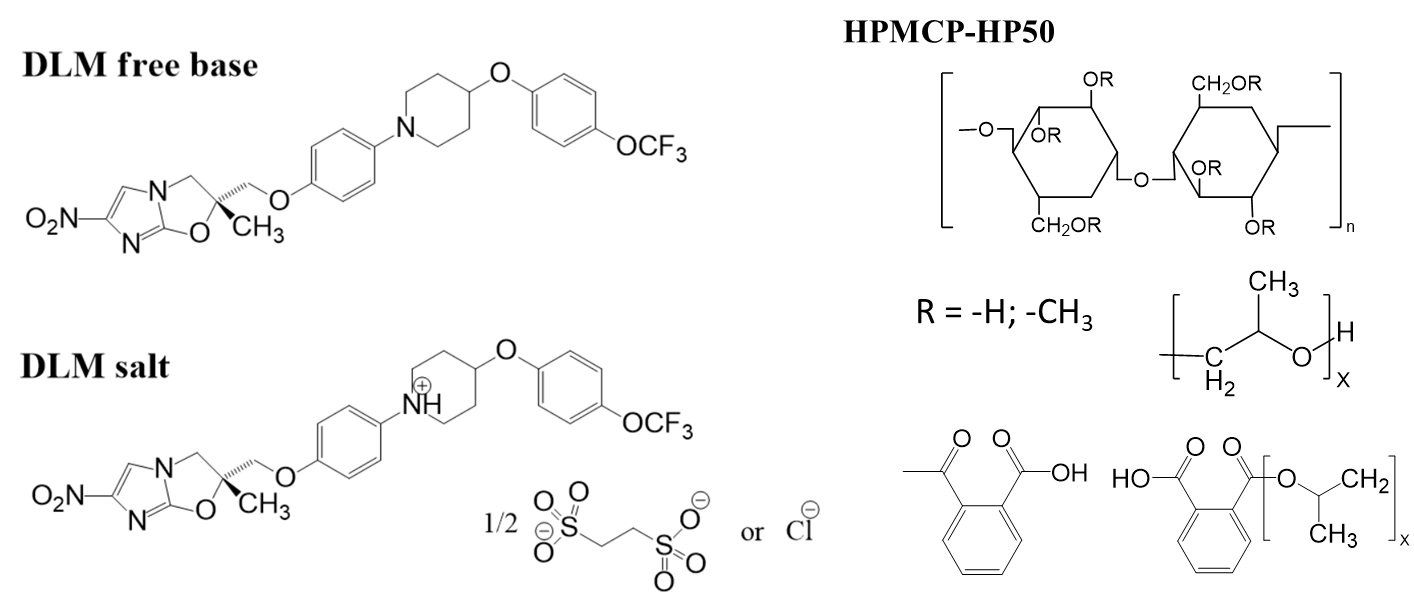


**Figure S8: Chemical structure of delamanid with pK_a_ of 4.3 (Shimokawa et al., 2015) in free base or salt form, and HPMCP-50**

**References**

Colorcon, 2014. Product lnformation on Acryl-EZE® II Optimized Aqueous Acrylic Enteric System. Colorcon.

EMA, 2013. Assessment report: Deltyba International non-propietary name: delamanid. European Medicines Agency.

Shimokawa, Y., Sasahara, K., Koyama, N., Kitano, K., Shibata, M., Yoda, N., Umehara, K., 2015. Metabolic Mechanism of Delamanid, a New Anti-Tuberculosis Drug, in Human Plasma. Drug. Metab. Dispos. 43, 1277-1283.

Van Duong, T., Nguyen, H.T., Taylor, L.S., 2022. Combining enabling formulation strategies to generate supersaturated solutions of delamanid: in situ salt formation during amorphous solid dispersion fabrication for more robust release profiles. European Journal of Pharmaceutics and Biopharmaceutics 174, 131-143.
